# Migrant workers' health-related research in Nepal: A bibliometric study

**DOI:** 10.1016/j.dialog.2023.100147

**Published:** 2023-07-20

**Authors:** Sharada P. Wasti, Ayushka Shrestha, Madhu Sudhan Atteraya, Vijay S. GC

**Affiliations:** a School of Human Sciences, University of Greenwich, London, United Kingdom; b Nepal Disabled Women Association, Kathmandu, Nepal; c Department of Social Welfare, College of Social Science, Keimyung University, South Korea; d School of Human and Health Sciences, University of Huddersfield, Huddersfield, United Kingdom

**Keywords:** Migration, Migrant-workers, Health, Bibliometric Analysis, Nepal

## Abstract

**Background:**

In recent years, the health of migrants has become an important global public health issue. However, less is known about the current status of research activity among Nepalese migrants' health. This study aimed to assess the current status of research activity by analysing published peer review literature on Nepalese migrants' health.

**Methods:**

A systematic search of published literature on Nepalese migrant workers' health was conducted in Scopus, Medline, CINAHL, Embase, PsycINFO and Web of Science, and a bibliometric analysis methodology was used. The search of databases retrieved 520 records, and a total of 161 papers were included in the analysis. Bibliometric analyses were performed in R and VoSViewer to create visualisation maps.

**Results:**

The retrieved documents were published in the last three decades, and a total of 533 researchers originating from 24 countries contributed to the literature. A large proportion of papers (n=22) were published in a single year, in 2019, and the number of authors per journal ranged from one to 14. The topmost preferred journals for publications in Nepalese migrants’ health were PLoS One (n=9), followed by the Journal of Immigration and Minority Health (n=6). The retrieved articles received 2425 citations, with an average of 15.1 citations per article. The study identified nine overlapping research domains (thematic areas) - infectious disease, non-communicable diseases, health and lifestyle, sexual and reproductive health, access to health services, workplace safety, maternal health, gender-based violence, and health system and policy.

**Conclusion:**

The present bibliometric study fills an analytical gap in the field of migrat's health research in Nepal and provides evidence and insights to advocate the formulation of strategies to promote the migrants' health vulnerabilities often associated with individual-related hazards such as working in 'difficult, dirty, and dangerous (3Ds) working conditions.

## 1 Introduction

The United Nations (UN) Sustainable Development Goals (SDGs) determine migration as a catalyst for development and recommend that "no one should be left behind for improving the health and human rights of migrants" [1]. The UN estimated that there were around 281 million international migrants in 2020, 3.6% of the global population, with nearly two-thirds being labour migrants [2]. Nepal is a lower-middle-income country where one in seven (approximately 3.8 million) Nepalese, excluding migrant workers in India, work abroad [3]. A recent national survey reported that nearly half (47%) of the households had at least one family member migrated either to internal or international destinations [4]. The migration outflow predominantly consists of low-skilled workers in the middle-east and Malaysia [5].

Migration is one of the determinants of health and is increasingly being recognised as a fundamental global health agenda [6]. The migration process, outcome, effects, and overall migratory circumstances have both positive and negative impacts on health outcomes. These have been studied from an interdisciplinary perspective dealing with diverse fields, including economics, sociology, health, and demography [7,8]. The economic and social aspects of migration, such as the importance of remittances on poverty reduction, development, socio-economic transformation, acculturation and integration issues, have been well-documented [[9], [10], [11], [12], [13], [14]]. Likewise, migrant workers' health, health outcomes, health needs, and availability and accessibility of health and wellbeing-related services have also been documented in country-specific contexts [15].

The relationship between migration and health is complex, and its impact varies considerably across migrant workers. For example, migrant workers from impoverished backgrounds are more likely to experience difficult life situations in the host country. They have had a lower level of physical and mental health as well as occupational vulnerabilities while working abroad [16,17]. Vulnerabilities were also associated with individual work-related hazards. The literature shows that Nepalese migrants predominantly occupy the lowest tier of manual labour, engaging in difficult, dirty, and dangerous (referred to as the 3Ds) occupations that local workers tend to avoid [18]. These sorts of working conditions often increase migrant’s health risks at the workplace [[18], [19], [20]]. Moreover, recent studies showed an increasing trend of Nepalese migrant workers dying abroad due to work-related vulnerabilities; nevertheless, a significant number of such deaths are unexplained [15].

Previous bibliometric analysis was performed in global migration health [21]. However, the review did not include studies reporting Nepalese migrants’ health. Furthermore, despite the growing number of Nepalese migrants over the past decade and increasing attention on their health and wellbeing [[22], [23], [24]], there has not been any assessment on mapping and examining peer-review literature about Nepalese migrants and their health. Such exploration would help explore the research and policy agenda to improve the health of Nepalese migrant populations. Therefore, this study aimed to present bibliometric indicators of published literature pertaining to Nepalese migrants. Specifically, this study examines the growth of publications, the geographical distribution of the publications, authorships, highly cited articles, international research collaborations, major research themes discussed and journals with the most publications.

## 2 Methodology

### 2.1 Data source and search strategy

The bibliometric method provides useful information on research publications' growth, impact, gaps and trends within a particular field or discipline [21,25]. Bibliometric scrutiny was performed to analyse the bibliographic characteristics of the included studies using six major electronic databases viz Scopus, Medline, CINAHL, Embase, PsycINFO, and Web of Science up to 30th December 2021. The methodology used in this research was similar to other bibliometric studies in this area [21,26]. The databases were searched comprising combinations of MeSH terms using keywords "migration", "migrant worker", "migrant", "left-behind" “health”, “wellbeing”, “wellness”, “mental health” and 'Nepal'. The detailed search strategy is provided in Appendix A. The search aimed to identify all the potential articles regardless of any health outcome related to migration and health in Nepal. The migration and health search queries were then applied to the title, abstract, and keywords of the publications.

### 2.2 Inclusion and exclusion criteria

Inclusion criteria were: i) Studies that examine the healthcare, health status, access to health services, or health-related issues faced by migrant workers or the left-behind families in Nepal or abroad, ii) papers published in peer-reviewed journals, conferences and dissertations and iii) articles published in the English language.

The exclusion criteria were: i) studies that do not focus on migrant workers or the left-behind families’ health and wellbeing related issues; ii) other than peer-reviewed publications such as books, book chapters, and training manuals and iii) papers published in other than English language.

### 2.3 Data screening and selection criteria

The identified potential papers were imported to EndNote, and duplicates were removed. The remaining citations were then exported to Rayyan (https://rayyan.qcri.org), including the broad citation information such as bibliographic information, title, author names, abstract, and keywords. Two reviewers (SPW and AS) independently screened the papers against eligibility criteria. The reference list of potential papers was also checked, and articles that had not been included earlier were added to make this study as comprehensive as possible. The third researcher (VSG) screened a subset of papers and resolved any discrepancies between the first two reviewers for the final review.

After piloting, a standardised data extraction form was used to extract data from the included studies. The extracted information included the name of the research article, the name of the research journal and statistics (i.e., the number of authors, impact factor, citation counts), the country of the publication, international networks/collaborations of authors, and country/author affiliation.

### 2.4 Data analysis and visualisation

All the bibliometric information of included studies was exported to Excel for analysis and tabulation. The exported data were used to generate specific indicators such as annual growth of publications, most frequent author keywords to know research interest and research gaps, calculation of international research productivity and collaboration patterns, and most active journals (with impact factor [27] and the number of publications) and authors with their Scopus affiliation (countries and research institutions). Different methods of publication analysis have been utilised, such as publication counts, name of the journal and country of publication, number of authors collaborating, number of citations received and impact factor, as they are the most common indicators of visibility and easier to access. For authorship analysis, we adopted similar methods used by Sweileh et al.’s bibliometric analysis in the global migration health arena [21]. Bibliometric analyses were performed using the R package *bibliometrix* [28], and VoSViewer was used to create visualisation maps.

## 3 Results

### 3.1 General information on the retrived documents

**Fig. 1:**
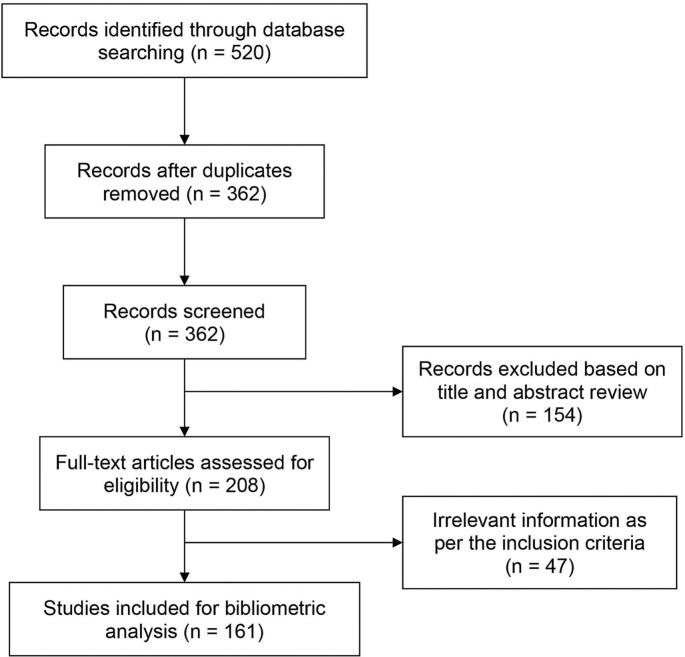
A scheme showing the general search strategy with the number of retrieved documents in each stage

The number of retrieved documents from the literature search is shown in Fig. 1. A total of 520 research papers were initially identified using the above-stated search criteria, and 312 papers were irrelevant after deduplication and title examinations. The full text of the 208 papers was then reviewed, and a total of 161 papers were included in the final analysis (Fig. 1).

Most papers (n=113, 70.2%) were published as primary research papers on migration health in Nepal, followed by secondary data analysis, narrative review, or systematic review papers (n=48, 29.8%). Over a third (n=58, 40.6%) of studies were conducted in Nepal, followed by Gulf Cooperation Council (GCC) countries (n=22, 15.4%) and Malaysia (n=12, 8.4%) respectively. Among the studies conducted in Nepal, a higher proportion of the studies (n=14) were carried out in more than one district in Nepal. Moreover, a majority of the study participants (73.9%) were migrant workers, followed by left-behind families (18%) and migrant women (3.1%) (Table 1).

**Table 1:** Study characteristics

Variables	Number	Percentage
Types of studies (N=161)		
Primary	113	70.2
Secondary data analysis	30	18.6
Review	18	11.2
Study conducted countries (n=143)		
Nepal	58	40.6
GCCs	22	15.4
Malaysia	12	8.4
United States of America	12	8.4
United Kingdom	10	7.0
Japan	8	5.6
Hong Kong	6	4.2
Australia	6	4.2
India	4	2.8
South Korea	3	2.1
Canada	2	1.4
Study conducted districts in Nepal (n=58)		
Multi-districts	14	24.1
Kathmandu	10	17.2
Doti	7	12.1
Achham	5	8.6
Chitwan	6	10.3
Kailali	3	5.2
Dhading	2	3.4
Baglung	2	3.4
Jhapa	2	3.4
Kanchanpur	2	3.4
Mustang	1	1.7
Nawalparasi	1	1.7
Palpa	1	1.7
Surkhet	1	1.7
Dolakha	1	1.7
Types of study participants (N=161)		
Migrant workers	119	73.9
Left behind families	29	18.0
Returnee migrants	8	5.0
Migrant women	5	3.1

### 3.2 Publication trends over the years

**Fig. 2:**
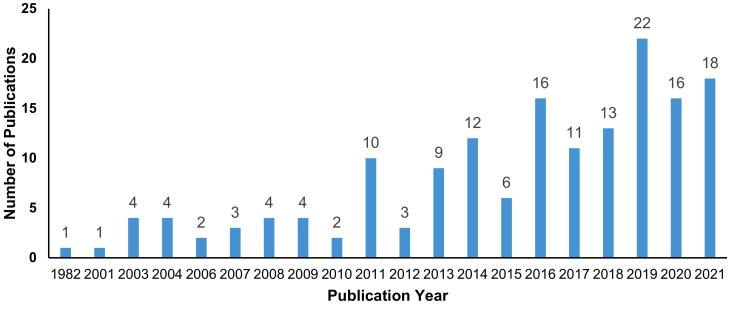
Publications trend over the year (N=161)

Over the last two decades, the number of publications on migration health in Nepal has increased substantially, showing an annual growth rate of 5.1 percent. The research on migration health in Nepal started in the early 1980s [29], and the number of published research articles began to increase during the 2000s. The peaks in publications were noticed in 2011, with the highest number of publications in 2019 (Fig. 2). The average age of the article was 7.3 years. The cumulative numbers show an upward trend in publications.

### 3.3 Terms and keywords analysis

**Fig. 3:**
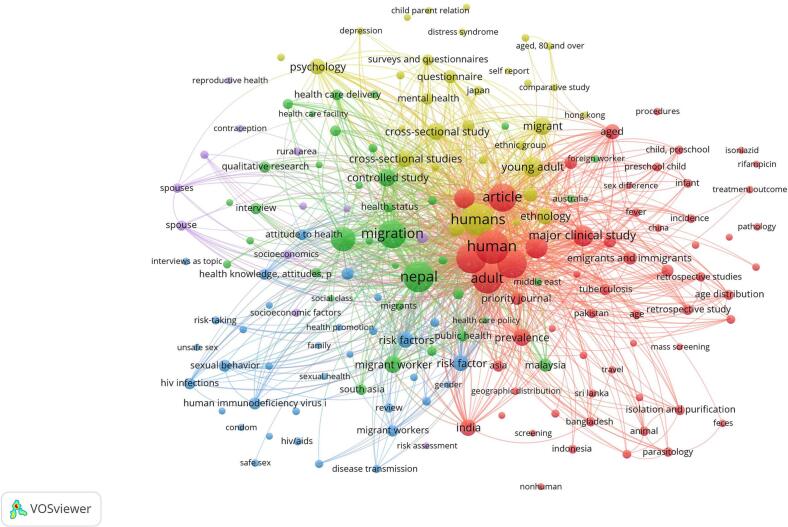
Network visualisation map of keywords in the literature pertaining to Nepalese migrant workers’ health

Fig. 3 highlights the co-occurrence of keywords which were the most used terms in the title and abstract fields of the included studies. A total of 1,337 keywords appeared in the 161 publications, whereas the author provided keywords were 383. The most frequently used keyword was Nepal, followed by migration and migrant workers (Fig. 3). These keywords may be helpful for academicians and authors to delve into the evolving literature on migration health in Nepal.

### 3.4 Total number of journals and citations

The retrieved documents were published in 119 peer-reviewed journals. The top five preferred journals for publication were PLoS One (9 papers), followed by the Journal of Immigration and Minority Health (8 papers), Parasites and Vectors (4 papers), and BMC Public Health (4 papers), respectively. The retrieved articles received 2425 citations, with an average of 15.1 citations per article (Table 2).

**Table 2:** Total number of journals and citations

Particulars	Number
Total number of journals	119
Top five journals	
PLoS One	9
Journal of Immigrant and Minority Health	6
International Journal of Environmental Research and Public Health	5
Parasites and Vectors	4
BMC Public Health	4
Total citation	2425
Average citations	15.1

### 3.5 Authorship analysis

Around two-thirds (65.8%) of the studies were conducted with multi-country collaboration. A total of 575 authors were identified, with an average of 3.8 authors (standard deviation (SD) = 2.2) per document. This value ranged from one to 14 authors per paper. A higher proportion of papers were written by four authors (29/161), whereas ten papers were single-authored. On average, there were 4.8 co-authors per document, and the authors' collaboration index was 3.7 (Table 3).

**Table 3:** Study collaboration and the number of authors per journal

	Number
Number of documents with multi-country collaboration (%)	106 (65.8)
Total number of authors	575
Mean number of authors (SD)	3.8 (2.2)
Single authored documents	10
Multi-authored documents	565
Co-authors per documents	4.8
Collaboration index	3.7
Publication with four authors	29

Table 4 shows the top five countries according to the lead author’s country of affiliation. The studies conducted on Nepalese migrants' health were predominantly conducted by scientists originating from the United Kingdom (n=52, 42.9%), followed by the USA (n=27), the Netherlands (n=11), Switzerland (n=8) and Germany (n=7). Other countries accounted for 13.2% of the total publications.

**Table 4:** Top five countries of lead authors' affiliation with the number of publications and citation

Ranking	Country	Records	Total citations
1	UK	52	1077
2	USA	27	250
3	Netherlands	11	471
4	Switzerland	8	51
5	Germany	7	117

**Fig. 4:**
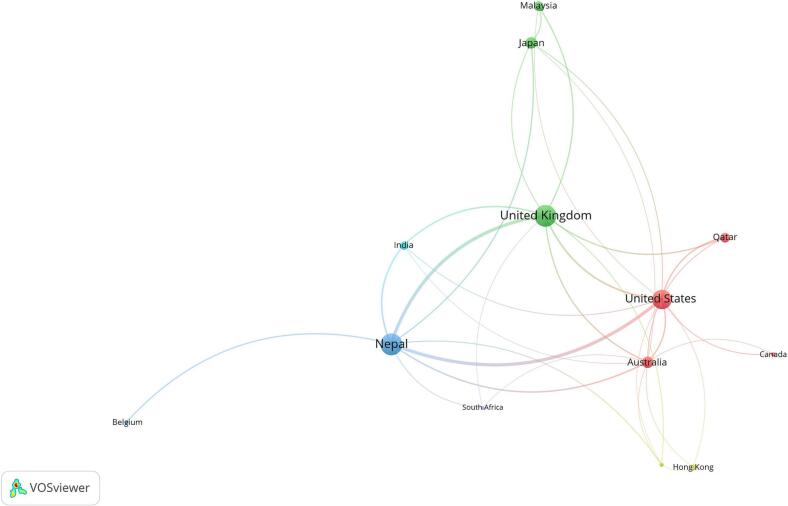
Collaboration among countries

The social map shown in Fig. 4 depicts the pattern of international collaboration between study authors, and at least three studies were produced among the countries.

While analysing the frequency of publications by active authors in the field, one author had published 18 papers. All the top three authors’ papers were published between 2008 to 2021. The top two contributors were from the UK, with the third from UK/Nepal (Table 5).

**Table 5:** Top five author's writing papers over time

Author name	Frequency	Publication Periods	Citation	H-Index⁎	Affiliation	Country
van Teijlingen, E	18	2008-2021	176	8	Health and Social Sciences, Bournemouth University	UK
Shimkada P	12	2008-2021	184	6	School of Human and Health Sciences, University of Huddersfield	UK
Adhikari P	11	2008-2021	133	6	Department of Public Health, University of Aberdeen; School of Health & Social Care, Bournemouth University; Green Tara Nepal	UK & Nepal
Regmi P	10	2016-2021	104	5	Bournemouth University.	UK
Aryal N	9	2016-2021	104	5	Faculty of Health and Social Sciences, Bournemouth University; University of Otago, Wellington	UK & New Zealand

⁎ Author's H-index in this table is only based on the included papers in this study.

The topmost cited country was the USA (n=493), with an average article citation of 20.5 followed by the UK (n=414, average article citation of 15.9), Japan (n=199, average citation of 18.1), and Australia (n=158, average citation 15.8) respectively. The overall average citation per document was found to be 13.2, and the average citation per year per document was 1.8 (Table 6).

**Table 6:** Top five most cited countries

Country	Total Citations	Average Article Citations
USA	493	20.5
United Kingdom	414	15.9
Japan	199	18.1
Australia	158	15.8
Qatar	123	13.7

### 3.6 Article citation analysis by journal

The top-cited paper was a systematic review that examined HIV risk among labour migrants [30]. The article published in the *AIDS and Behaviour Journal* was the highest-cited article (156 citations; 14.2 citations per year), followed by Clinical Cardiology (98 citations) respectively (Table 7).

**Table 7:** Top-five most-cited publications in migration and health in Nepal

Authors (Year)/Title	Journal	Total citations	Total citation per year
Weine SM & Kashuba, AB (2012) Labor migration and HIV risk: a systematic review of the literature	AIDS and Behaviour Journal	156	14.2
Kanaya AM et al. (2013) Mediators of atherosclerosis in South Asians Living in America (MASALA) study: objectives, methods, and cohort description	Clinical Cardiology	98	9. 8
Poudel KC et al. (2003) Mumbai disease in far western Nepal: HIV infection and syphilis among male migrant-returnees and non-migrants	Tropical Medicine and International Health	69	3.5
Lyu, S et al. (2018) Relationships among safety climate, safety behaviour, and safety outcomes for ethnic minority construction workers	International Journal of Environmental Research and Public Health	66	13.2
Turner-Moss E, et al. (2014) Labour exploitation and health: a case series of men and women seeking post-trafficking services	Journal of Immigration and Minor Health	64	7.1

While comparing the journals with their impact factor, the Lancet HIV had the highest impact factor (12.76) [31], followed by Chest (9.66) [32], Obstetrics and Gynaecology (4.97) [33], PLoS Neglected Tropical Disease (4.49) [34] and Journal of Travel Medicine (4.16) [35] respectively.

### 3.7 Research domains

**Fig. 5:**
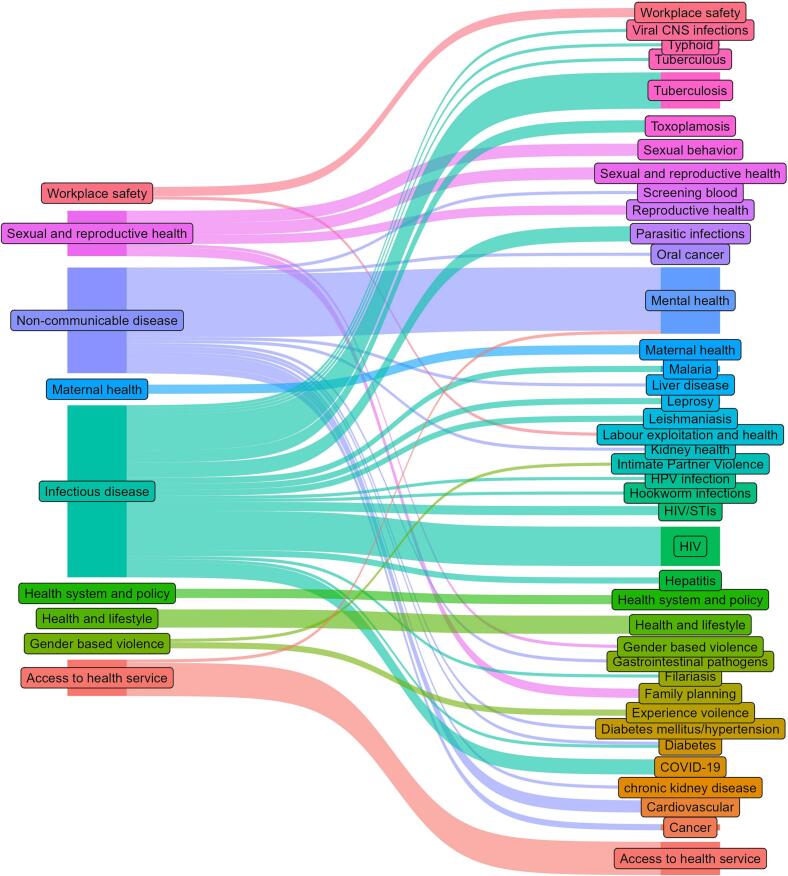
Key migration health-related themes

In the study, a total of nine overlapping research domains (thematic areas) were identified. Among the major themes, infectious disease-related issues were the most common (n=57, 35.4%), followed by non-communicable diseases (n=35, 23.6%), sexual and reproductive health (n=17, 10.6%), health and lifestyle (n=17, 10.6%), access to health services (n=15, 9.3%), workplace safety (n=8, 5%), maternal health (n=3, 1.9%), gender-based violence (n=3, 1.9%) and health system and policy (n=3, 1.9%) respectively. Similarly, a higher proportion of research was published specifically on mental health issues (n=22) followed by HIV/AIDS, including Sexual Transmission Infections (n=16), sexual reproductive health (n=15), Tuberculosis (n=14) and access to health services (n=11) respectively (Fig. 5).

## 4 Discussion

Our study identified a growth in research on migration health in Nepal and increased importance of research on Nepalese migrants' health. Findings depicted a positive growth in scientific production over the last two decades, but a significant increase can be seen between 2011 and 2021. This might be related to Nepal’s increased dependence on exporting labour migrants in the global market and increased demand of labour jobs in the global market in the last decade [36]. The significant finding was that collaboration between authors and countries seemed to be led by the United Kingdom, where the top four authors were based. However, the global migration health research bibliometric study found that the USA ranked first, followed by the UK [21].

Over the last two decades, Malaysia and the countries in the Middle East have become large receivers of Nepalese migrants. The top 20 largest receivers of Nepalese temporary labour migrants belong to Malaysia and the countries in the Middle East [37]. There are more Nepalese migrants abroad today than ever, which is an increasingly important contributing factor to Nepal’s economy, where remittance contributes around one-third (26.9%) of Nepal’s gross domestic product [5]. This clearly shows that many migrants choose to live abroad to improve the lives of their immediate and left-behind families. Similarly, there were increasing publications related to Nepalese migrants’ health. The first review paper in this area examined HIV risk among labour migrants and was the top-cited paper in this review [30]. This depicts that infectious disease particularly HIV/AIDS was highly prevalent among migrant workers in India and connotated as a “Mumbai disease” [38,39].

Writing and choosing the most appropriate keywords in peer review journals is crucial as it allows accurate manuscript indexing in bibliographic databases and allows researchers to locate the publication efficiently. Three kinds of keywords - a word in the title, author keywords and keywords plus were analysed, and all sources could not find similarities with each other. The meaning of the single words in titles sometimes does not make sense and needs referring to the authors' keywords for better understanding. However, the authors’ keywords were very generic and often did not match with papers. The reason for varied keywords might be that the research area is thus a relatively new field undergoing continual development and evolution. Therefore, migration health researchers should be cautious while providing author keywords for their manuscript, which should reflect the thematic research areas.

The retrieved papers were published in 119 different peer-reviewed journals. Only five journals were related to migration or migration health, and only nine papers were published in these journals. Surprisingly the first publication in 1982 was in the Journal of International Migration Review [29]. All those five migration-related journals were published internationally, and not a single journal was run nationally. Likewise, the top five journals with higher numbers of publications and high impact factors publications were in the non-migration-related field. Previous reviews on global migration health reported the Journal of Immigrant and Minority Health as the topmost journal. Furthermore, among the top ten preferred journals, five were in the field of migration [21].

Next, not all journals were listed for journal citation reports – more than 20 papers were published in a journal without an impact factor. The highest impact factor journal was the Journal of Chest (IF 16.9) [31], followed by the Southeast Asian Journal of Tropical Medicine and Public Health (IF 0.29) [40], one of the lowest impact factor journals in the list. Surprisingly, the topmost publication journal was not specific to migration-related fields but was published in the crosscutting thematic areas of general health, HIV/AIDS, Obstetrics and Gynaecology, Tropical Medicine and Travel Medicine [15,31,33,34].

Among the nine overlapping health and well-being-related research domains, infectious disease-related issues appeared in 57 publications, followed by non-communicable diseases. The reason behind much focus on infectious diseases could be that migrant workers in South Asia generally seem to have a greater prevalence of infectious diseases due to the complex interaction of several factors, including poor access to healthcare and low socio-economic status [18]. Likewise, mental health-related issues had more publications than the other single thematic issues. The higher number of research in this thematic area might have been that the migration outflow consists predominantly of low-skilled workers who are often employed in occupations considered ‘difficult, dirty and dangerous’ (3Ds) [19]. However, the global migration health bibliometric analysis illustrates that psychosocial and mental health was the leading theme, followed by infectious disease [21]. The utilisation of health services, including health insurance coverage, injuries and physical health-related issues, including exposure to workplace violence, abuse, job satisfaction and workers' quality of life, are key areas for further research. Study shows that a large number of returnee migrants were debilitating injuries and both mental and physical illness [20,41]. Likewise, sexual and reproductive health, including maternal health services, constitutes a small proportion of research publications, even though 8.5% of Nepalese women are international labour migrants [37].

## 5 Strengths and limitations of the study

To our knowledge, this is the first bibliometric study on migration health in Nepal. This study used a systematic approach to search, screen and synthesise the literature, covering literature published in the field since the early 1980s. Our results are based only on publications in academic journals. This study has used six prominent databases to capture all the publications published in peer-reviewed journals, which could have been beneficial to study the topic. Given the weight placed in academia on publishing in peer review journals, these findings are important to consider. Furthermore, our results likely allude to the global dynamic within migration health research in Nepal. Some limitations constrain our study; firstly, in terms of the study design, we only focused on articles, reviews and editorials that have been published in peer-reviewed journals. Although our database search was comprehensive, some peer-reviewed journals might not have been indexed in these databases, especially those published in Low and Middle-Income Countries. Secondly, this study did not consider grey literature (e.g. books, thesis and report), negatively affecting the total number of retrieved documents. Thirdly, we could not assess the quality of the included research publications because the included papers could be pivotal in a subject and significantly impact the credential of the discipline. But all the included papers were published in peer review journals. Hence this bibliometric study presents a valid picture of published research activity in migration health in Nepal. Another limitation is the inclusion of only English-language articles, whereas some Nepali publications may exist. Lastly, this review did not include book chapters and conference papers which could also be a probable limitation.

## 6 Conclusions

This study showed that Nepalese migrant health research is a key research topic in the global health arena, where one author published 13 publications on Nepalese migrant health issues in peer-reviewed journals. Findings highlight the significant number of publications, citations, leading authors and journals and keywords co-occurrence to summarise the literature. Most articles were published about infectious disease-related issues, followed by health and lifestyle, sexual and reproductive health, access to health services, workplace safety, maternal health, and health system and policy. Nevertheless, this study provides researchers of migration health with valuable information to help them better identify potential collaborators, current hotspots and future research directions, including the need for the involvement of migrants in health research [42]. The finding also guides the authors where the Nepalese migrant health research obtained the highest number of publications that guides future researchers to correctly choose the right journals to publish their research to obtain greater visibility.

## Funding

This research received no external funding.

## Authors’ contributions

SPW and VSG designed the study, searched the literature and wrote the first draft of the manuscript. AS and MA screened the articles, extracted the data, carried out the quality assessment and contributed to the initial drafts. All authors reviewed and edited the manuscript and approved the final version of the manuscript.

## Declaration of Competing Interest

The authors declare no conflicts of interest.

## Appendix A Supplementary data

Supplementary material Image 1
