# Supplementary material for: Migrant workers' health-related research in Nepal: A bibliometric study
Source: Dialogues Health. 2023 Jul 20;3:100147. doi: 10.1016/j.dialog.2023.100147 (PMC10954020; doi:10.1016/j.dialog.2023.100147)
Supplement: Supplementary file 1 — Supplementary material [file mmc1.docx]

# **Search strategy:**

**Medline, Embase and PsycInfo via OvidSP**

1 migrant.mp.

2 exp *Human Migration/

3 migration, human/

4 (minority adj2 group*).mp.

5 refuge*.mp.

6 "Emigration and Immigration"/ or Refugees/

7 asylum.mp.

8 1 or 2 or 3 or 4 or 5 or 6 or 7

9 *Health/

10 *disease/

11 wellbeing.mp.

12 Health Status/

13 wellness.mp.

14 9 or 10 or 11 or 12 or 13

15 Nepal.mp. or *Nepal/

16 nepali.mp

17 15 or 16

18 8 and 14 and 17

**Web of science**

#1 TS = (migration or migrant* or immigra* or (minority near groups) or refuge* or asylum or left behind)

#2 TS = (health or medical or disease* or wellbeing or wellness)

#3 TS = (Nepal or Nepalese or Nepali)

#4 #1 AND #2 AND#3

**Scopus**

TITLE-ABS-KEY ( migration OR migrant* OR immigra* OR ( minority AND near AND groups ) OR refuge* OR asylum OR left behind) AND TITLE-ABS-KEY ( health OR medical OR disease* OR wellbeing OR wellness ) AND TITLE-ABS ( nepal OR nepalese OR nepali ) AND ( LIMIT-TO ( DOCTYPE , "ar" ) ) AND ( LIMIT-TO ( LANGUAGE , "English" ) )

**CINAHL**

MH ( migration or migrant* or immigra* or emigration or refuge* or asylum or left behind) AND TX ( health or wellbeing or well being or well-being or quality of life or disease* or wellness ) AND MW ( nepal or nepali or nepalese )
